# Suction Impact on Chest Drain Duration After Lung Resection: A Study Using Digital Drainage Systems

**DOI:** 10.3390/jcm15103592

**Published:** 2026-05-08

**Authors:** Dan Levy Faber, Juna Azizi, Ronen Galili, Sonia Schneer, Abed Agbarya

**Affiliations:** 1 Department of Cardiothoracic Surgery, Lady Davis Carmel Medical Center, 7 Michal Street, Haifa 3436212, Israel; danfaber1@hotmail.com (D.L.F.); junaaz@clalit.org.il (J.A.); ronenga@clalit.org.il (R.G.); soniaschneer@gmail.com (S.S.); 2 The Ruth and Bruce Rappaport Faculty of Medicine, Technion, Israel Institute of Technology, 1 Efron Street, Haifa 3339101, Israel; 3 Department of Oncology, Bnai-Zion Medical Center, 47 Eliyahu Golomb Avenue, Haifa 3339419, Israel

**Keywords:** lung resection, postoperative chest drain, suction, digital chest drainage system, prolonged air leakage, hospital length of stay

## Abstract

**Background/Objectives**: The aim of this study was to evaluate whether no suction management to chest drains after pulmonary resection shortens the duration of chest tube placement and improves postoperative outcomes compared to routine application of negative-pressure suction to chest drains. **Methods**: A single-center randomized controlled study was conducted in patients undergoing lung resection. Patients received a single 28Fr chest tube attached to a digital drainage system. Patients were randomized to Control (chest tube on continuous suction) or No Suction. **Results**: From December 2022 to April 2025, 309 patients were enrolled; 23 patients were excluded for protocol deviations. In total, 286 patients were analyzed (149 Control, 137 No Suction). Chest tube duration was shorter in the No Suction (mean 40.2 ± 43.8 h) than the Control group (53.6 ± 67.8 h, *p* = 0.002). Hospital length of stay and the incidence of prolonged air leak did not differ between No Suction and Control. In multivariable regression, suction was associated with a 13 h longer time to drain removal (95% confidence interval 0.03 to 27 h; *p* = 0.050), without a significant effect on length of stay or odds of prolonged air leak. Patients with underlying lung disease or undergoing anatomical resection had overall longer chest tube durations. **Conclusions**: Application of suction to chest tubes after lung resection prolongs the duration of chest drainage without improving clinical outcomes. Managing chest tubes with physiologic intrapleural pressure and no suction may allow for earlier removal of drains and should be considered as the approach in uncomplicated lung resections.

## 1. Introduction

Thoracic surgery plays a crucial role in the treatment of lung diseases, including lung carcinoma [1]. Anatomical lung resections dissect along defined anatomical planes (e.g., pneumonectomy, lobectomy, and anatomical segmentectomy). Non-anatomical lung resections (wedge resections or non-anatomical segmentectomies) remove a portion of lung tissue regardless of segmental boundaries [2]. Pulmonary resection requires proper postoperative management of the pleural space to ensure lung re-expansion and to prevent complications from air or fluid accumulation [3].

Chest tube/intercostal drain (ICD) thoracostomy is the procedure of choice for pleural drainage for postoperative thoracic surgery patients [4,5], used for evacuating air (pneumothorax) and fluid (blood, effusion) from the pleural cavity and for reestablishing the negative intrathoracic pressure that is essential for lung expansion. The ICD also serves as a diagnostic monitor for air leaks [6].

In a traditional water-seal chest drainage system, the intrapleural pressure varies with respiration, and air/fluid exit into a collection chamber through a column of water that prevents backflow [7,8]. A digital chest drainage system (DDS) employs electronic sensors and an integrated suction/monitoring unit to continuously measure intrapleural pressure and quantify air leak flow rates (mL/min) [5,9]. DDS may reduce chest tube duration (CTD) and length of stay (LOS) compared to traditional analog systems [10]. Additional negative-pressure suction can be applied to both ICD systems [9] to further lower pleural pressure and expedite lung re-expansion by actively drawing air and fluid out of the pleural space [6]. Suction should help oppose the visceral and parietal pleura, thereby promoting sealing of any alveolar air leaks and eliminating residual dead space after resection [11]. There is a debate about whether suction is beneficial or necessary in all cases [12]. Critics of suction note that it increases airflow through the chest tube, which can exacerbate the apparent size of an air leak and potentially impede leak closure [9].

Higher negative intrapleural pressures might pull air through a marginal air leak that might otherwise close on water seal, thereby prolonging the duration of the leak [13]. However, placing the tube to water seal (without suction) in the presence of a large air leak can risk accumulation of air in the pleural space or inadequate drainage of fluid, if the lung is not fully re-expanded [14]. Thus, some surgeons selectively employ suction in certain scenarios (e.g., large air leaks or significant pleural space) [15].

A common complication following lung resection is a prolonged air leak (PAL) lasting > 5 days [16,17]. PALs are associated with increased patient morbidity, pain from prolonged CTD, higher infection risk, and extended hospitalization. Reported incidence of PAL are 8–15% of patients, with higher rates in those with underlying lung disease [18]. Risk factors for PAL include underlying pulmonary conditions (chronic obstructive pulmonary disease (COPD) or emphysema), the extent and type of resection (anatomic resections, lobectomy, carry higher risk than wedge resections), older age, male gender, low body mass index (BMI), smoking history, and prior thoracic surgery [19,20]. Many of these factors reflect reduced pulmonary reserve or more complex surgery, which predispose the patient to an air leak [21,22].

Some trials suggested that keeping ICD on water seal might shorten air leak duration and CTD compared to suction, whereas others found no significant difference between suction vs. no suction management [14,23,24,25,26,27]. Systematic reviews concluded that there was no advantage of routine suction over water seal, no significant differences in PAL incidence, total drainage duration, or hospital stay between external suction and water seal management [28,29]. The 2019 Enhanced Recovery After Surgery Society (ERAS) and European Society of Thoracic Surgeons (ESTS) guidelines recommend ICD be placed to water seal unless specific indications arise [9]. STS released an Expert Consensus Statement (2024) on pleural drain management after lobectomy, and suggest that for routine cases, gravity drainage (water seal) is sufficient, with suction reserved for selected situations such as significant air leak [17]. Despite these recommendations, practice remains variable and often depends on surgeon preference.

At Carmel Medical Center (CMC), prior to this study, the routine protocol for lung resections was to apply −20 cmH_2_O suction to the ICD immediately after surgery through the first postoperative night (POD 0), then switch the system to no suction physiologic −8 cmH_2_O pressure on the DDS on the morning of POD 1. Chest tube removal (CTR) was decided based on the cessation of the air leak as indicated by the digital flow readings (no events of >20 mL/min air leak for >6 h) and acceptable fluid output <200 mL per 24 h. CTR was done only during the morning or evening shifts. The current study hypothesis was that omitting routine initial suction might allow earlier cessation of air leaks and shorten the time to CTR, without affecting patient outcomes. The aim of this study was to compare outcomes between Suction vs. No Suction ICD management, using objective DDS monitoring to accurately measure air leak and fluid outputs. The primary endpoint was CTD, with secondary endpoints of PAL incidence and hospitalization LOS, as these factors impact patient recovery and healthcare utilization.

## 2. Materials and Methods

### 2.1. Study Design and Randomization

This single-center study was conducted in accordance with the Declaration of Helsinki, and approved by the Institutional Review Board of CMC (CMC-0087-22 on 2 August 2022). Informed written consent was obtained from all participants. During the preparation of this manuscript, the authors used the generative-AI assistant Claude 3 (Anthropic, San Francisco, CA, USA) to assist with English-language editing, supportive literature search, and auxiliary review of the R code used for the statistical analyses. All statistical analyses were performed by the authors in R version 4.4.1, and all numerical results were verified independently against the source dataset. No AI tool had access to identifiable patient data. The authors reviewed and edited every AI-derived suggestion and take full responsibility for the integrity and content of this publication.

Eligible participants were adult patients > 18 years, who underwent elective lung resection surgery (including wedge resection, anatomical segmentectomy, or lobectomy). Patients who underwent pneumonectomy were excluded from the trial due to differing ICD management. On surgery day, patients were randomized to postoperative ICD management strategies: No Suction (Intervention) vs. Suction (Control). A stratified randomization was employed based on factors associated with postoperative air leak duration and complications: (1) BMI (<22 vs. ≥22), (2) presence of chronic lung disease (excluding the lung cancer diagnosis), and (3) type of resection (anatomical vs. non-anatomical). Within each stratum combination, patients were randomly assigned in a 1:1 ratio to the two management groups.

### 2.2. Chest Tube Management Protocols

All patients had one 28 Fr silicone ICD inserted at the conclusion of the lung resection, placed in the ipsilateral pleural space through an intercostal incision. Each ICD was connected to a chest DDS (Thopaz™, Medela Healthcare, Baar, Switzerland). The Intervention group (No Suction) patients had their ICD set to physiologic intrapleural pressure (−8 cmH_2_O) on the DDS, without external suction. This replicates a traditional water-seal gravity drainage, allowing the device to record air leak and pleural pressure data. The Control group (Suction) patients had their ICD connected to continuous negative pressure at −20 cmH_2_O immediately after insertion and throughout the first postoperative night (Postoperative Day 0). On the morning of POD 1, suction was discontinued for Control patients; the chest drainage device was then set to −8 cmH_2_O. Thereafter, both groups were managed similarly on −8 cmH_2_O pressure. CTR criteria were: no events of >20 mL/min air leak for >6 h and acceptable fluid output of <200 mL per 24 h. If an air leak persisted, the tube was left in place regardless of POD until the leak ceased or was deemed prolonged, requiring intervention. The exclusion criteria from the trial analysis were predefined: I. if surgery was converted to pneumonectomy; II. if a patient in the No Suction group developed a large clinically significant air leak with signs of lung collapse and massive percutaneous emphysema, applying suction or adding an additional chest tube at the surgeon’s discretion; III. a patient with an acute complication needing a second surgical intervention; and IV. patients with PAL > 14 days that were discharged for ambulatory follow-up with the drainage system. The study was designed to focus on uneventful standard lung resection without major complications.

The threshold of an air-leak flow of ≤20 mL/min sustained for more than 6 h was selected a priori as a conservative, safety-oriented criterion. Reported thresholds in studies using digital drainage systems range from 20 to 50 mL/min for 4–12 h; lower flow cut-offs and longer observation windows reduce the risk of post-removal pneumothorax at the expense of slightly later chest tube removal. The choice of the lower end of this published range, applied uniformly across both study arms, was intended to minimize differential bias in the timing of removal and to allow safe earlier removal in patients without an ongoing leak.

### 2.3. Endpoints

The primary endpoint was time to CTR (hours from the end of surgery). Secondary endpoints were (1) PAL, and (2) postoperative hospital LOS days (discharge day was counted). Additional data collected included any intervention related to air leak (such as additional ICD, use of autologous blood patch or chemical pleurodesis, or re-operation for persistent air leak).

### 2.4. Sample Size Calculation

The sample size was calculated to detect an 8 h difference in mean time to CTR between the two groups. An 8 h difference (1 work shift) was considered the minimal clinically meaningful difference, as it could translate to a patient having their CTR earlier in the day and potentially being discharged earlier. Based on prior data, the standard deviation (SD) of time to CTR was estimated to be 24 h. Using a two-sided α = 0.05 and power (1–β) of 0.8, the calculation indicated that 143 patients per group would be required to detect an 8 h difference. To account for dropouts or protocol deviations (estimated 5–8%), the aim was to recruit approximately 300 patients total (150 per arm).

### 2.5. Statistical Analysis

Baseline patient characteristics and operative details were compared between groups using appropriate tests: Pearson’s chi-square or Fisher’s exact test for categorical variables and Wilcoxon rank-sum test for continuous variables. A two-sided *p* < 0.05 was considered statistically significant. The first analysis examined whether there were any significant differences in demographics, comorbidities, or surgical factors between the No Suction and Suction groups. Because the randomization (with stratification) yielded no significant differences in baseline gender, age, smoking status, pulmonary function, resection type, or other characteristics (all *p* > 0.05), no covariate adjustments were deemed necessary in the primary outcome analysis.

For the primary and secondary endpoints, regression modeling was utilized to estimate the effect of Suction vs. No Suction. Linear regression was used for the continuous outcomes (hours to drain removal, and PODs to discharge), providing an estimated beta coefficient (mean difference) between the Suction and No Suction groups. Logistic regression was used for the binary outcome of PAL, reporting an odds ratio (OR) for the effect of suction. For each outcome, an unadjusted model with the group assignment as the predictor was fit. Pre-specified subgroup analyses were performed to explore whether the effect of suction was modified by the stratification factors (history of lung disease, low BMI, or anatomical resection). This was done by adding an interaction term between group and the factor of interest in the regression models. For example, to assess modification by lung disease, we included terms for group, lung disease, and group × lung disease interaction in the model. A significant interaction (*p* < 0.05) would indicate the effect of suction differs in patients with vs. without that factor. All statistical analyses were conducted using R version 4.4.1 (R Foundation for Statistical Computing, Vienna, Austria).

Because the distribution of time to chest tube removal was markedly right-skewed, the regression model for this endpoint was based on the natural-logarithm-transformed outcome rather than the untransformed scale; the exponentiated coefficient for the suction term is, therefore, reported as a ratio of geometric means with its 95% confidence interval. For length of stay, also right-skewed, a generalized linear model with a gamma distribution and a log link was used, and the exponentiated coefficient is reported as a multiplicative factor. For the binary endpoint of prolonged air leak, multivariable logistic regression was used and an odds ratio with its 95% confidence interval is reported. The Wilcoxon rank-sum test continued to be used for the unadjusted between-group comparison of continuous endpoints, in line with the right-skewed distribution of the data.

To address the possibility of residual confounding by measured covariates, pre-specified-as-sensitivity multivariable models were fitted for all three endpoints, additionally adjusting for age, sex, pack-years of smoking, and the three stratification factors (previous lung disease, BMI < 22 kg/m^2^, anatomical resection). The adjusted estimates are reported alongside the unadjusted ones.

The principal analysis was performed in the per-protocol population, defined as all randomized patients managed throughout the postoperative period according to their assigned strategy. As a pre-specified sensitivity analysis, an intention-to-treat (ITT) analysis was performed in which all randomized patients (*n* = 309) were analysed according to their original allocation, regardless of subsequent cross-over, escalation of treatment, conversion to pneumonectomy, reoperation, or ambulatory discharge with the drainage system. Both per-protocol and ITT estimates are reported, with the ITT analysis treated as the principal robustness check on the conclusions.

## 3. Results

### 3.1. Patient Enrollment and Exclusions

Between 12 December 2022 and 7 April 2025, a total of 309 patients undergoing lung resection at CMC were enrolled and randomized. There were 23 patients that were excluded due to pre-specified criteria or protocol deviations. In total, 286 patients were analyzed, 149 patients in the Control (Suction) and 137 in the No Suction group (Table 1).

The 23 excluded patients comprised: 5 patients in the No Suction arm who developed a clinically significant air leak with lung collapse and/or massive subcutaneous emphysema and were converted to suction or received an additional drain at the surgeon’s discretion; 4 patients whose planned resection was converted intraoperatively to pneumonectomy; 8 patients who experienced an acute complication requiring a second surgical intervention; and 6 patients with prolonged air leak > 14 days who were discharged for ambulatory follow-up with the digital drainage system.

### 3.2. Baseline Characteristics

The Control and No Suction groups were balanced in baseline demographic and clinical characteristics (Table 1), without statistically significant differences in age, gender distribution, smoking history, or indication for surgery.

### 3.3. Operative Details

The types of lung resection performed were similarly distributed between the two study arms (Table 2) without significant differences, hence eliminating surgery extent as a confounder in comparing air leak duration.

The proportion of wedge vs. lobectomy vs. segmentectomy cases did not differ significantly between the No Suction and Control groups (*p* > 0.4 for all comparisons). All procedures in this study were performed using a minimally invasive video-assisted thoracoscopic surgery (VATS) approach; no open thoracotomies were included. The exclusive use of VATS eliminates surgical-approach heterogeneity as a potential source of variability in postoperative outcomes.

All stratification variables were balanced between groups (by design), without statistically significant differences in the proportion of patients with prior lung disease, low BMI, or undergoing anatomical surgery (Table 3).

### 3.4. Primary Outcome—Time to Chest Tube Removal

The intention-to-treat (ITT) sensitivity analysis described in Section 2.5, which retains all 309 randomized patients in their original allocation arm, yielded results consistent in direction with the per-protocol analysis, with an attenuated but still favorable effect of No Suction on time to chest tube removal; the ITT results are reported alongside the per-protocol estimates in Table 4 and Table 5.

The primary outcome of CTD was significantly shorter in the No Suction compared to the Control group (40.2 vs. 53.6 h, *p* = 0.002) (Table 4). Patients without Suction had CTR mean of 13 h earlier than those in the Suction group. The variance was large (evidenced by the SDs and range) due to a subset of patients with PAL in each group. No delays in chest tube removal were attributed to pleural fluid accumulation.

### 3.5. Secondary Outcomes

The secondary endpoints, postoperative hospital LOS and PAL incidence did not differ statistically between the Suction and No Suction strategies (Table 4). Nearly all patients were discharged by POD 3 or 4. No patient in either group developed a tension pneumothorax or clinically significant undrained hemothorax due to omission of suction.

### 3.6. Multivariable Analysis

Regression analyses were performed to quantify the independent effect of suction on each outcome while accounting for variability. Table 5 confirms that suction significantly prolongs CTD by 13 h, independent of other factors. There was no significant effect of suction on LOS or the probability of a PAL in this analysis, reinforcing that the primary impact of suction was to delay CTR. The confidence interval for the suction effect on CTD barely excludes 0 (lower bound 0.03 h), corresponding to the *p* = 0.05 threshold of significance. This finding is clinically coherent with the group comparison above. The odds ratio > 2 for PAL with suction suggests a possible tendency towards more PAL in the suction group.

### 3.7. Subgroup and Interaction Analyses

Further analysis explored whether the effect of suction was consistent across various subgroups defined by the stratification factors: presence of previous lung disease, low BMI, and anatomical vs. wedge resection (Table 6).

### 3.8. Effect Modification by Previous Lung Disease

The regression analysis indicates that having a lung disease history was associated with a longer CTD (β + 22 h vs. no lung disease, *p* = 0.063) and significantly higher odds of PAL (OR 6.4, *p* = 0.048). However, the interaction term between suction use and lung disease was non-significant for any endpoint (interaction *p* = 0.4 drain time, *p* = 0.5 LOS, *p* = 0.6 PAL). The impact of omitting suction was similar in patients with and without lung disease (Table 6). The lack of a significant interaction suggests that suction offers no special benefit in patients with compromised lungs, and their likelihood of PAL was driven by their disease rather than mitigated by suction.

### 3.9. Effect Modification by BMI

Low BMI was not a significant predictor of PAL or other outcomes in this study (Table 6), implying that even in lean patients, suction did not confer advantage in resolving air leaks.

### 3.10. Effect Modification by Resection Type

An anatomical resection was associated with an increase of 28 h in drain time (*p* = 0.004) and 1.7 days longer LOS (*p* < 0.001) compared to a wedge resection, reflecting the greater surgical extent and tissue dissection (Table 6). Anatomical resection did not significantly increase the odds of a PAL in this study (OR 4.0, *p* = 0.2, possibly due to limited PAL events). However, the interaction between suction and resection type was not significant for any outcome (all interaction *p* > 0.1). The effect of suction (prolonging CTD) was present in both subgroups and appeared slightly attenuated in the anatomical resection group (interaction β = −17 h for drain time, *p* = 0.2, indicating a non-significant trend that suction might have slightly less relative impact in lobectomy patients). Both wedge and anatomical resection patients had shorter CTD without routine suction.

Overall, undergoing an anatomical resection (lobectomy/segmentectomy) was associated with significantly longer CTD and LOS compared to a wedge resection, regardless of ICD management (*p* < 0.01). The benefit of omitting suction (shorter CTD) was observed in both wedges and lobectomies, without statistically a significant interaction. In this study, suction did not improve outcomes in any patient subgroup. The factors of chronic lung disease and extensive resection were associated with longer CTD overall and longer CTD with suction (though one might hypothesize suction could have been helpful in non-expanding lungs, the present data do not support routine use in that context).

The primary hypothesis of the study was confirmed, omitting routine postoperative suction resulted in a significantly shorter time to CTR. The incidence of PAL was low and similar between groups, suggesting that not using suction did not increase the risk of clinically significant persistent air leak.

## 4. Discussion

This randomized study was designed to address a question in postoperative thoracic surgery management: does routine application of wall suction to ICD after lung resection confer any advantage, or could it be safely avoided to facilitate faster recovery? [16,17,21,22]. The main finding is that use of suction after pulmonary resection prolongs the CTD by half a day and offers no benefits in terms of air leak cessation or hospital stay.

From a physiological standpoint, external suction increases the magnitude of the alveolar–pleural pressure gradient. Because the alveolar pressure during expiration approximates atmospheric pressure while the pleural pressure becomes substantially more negative under −20 cmH_2_O suction, the trans-pulmonary pressure across any small parenchymal defect is amplified. This increased gradient may drive air through micro-fistulas that would otherwise close spontaneously when the lung is allowed to remain at physiologic intrapleural pressure (approximately −8 cmH_2_O). In addition, sustained negative pleural pressure increases visceral-pleural mechanical stress, which may prolong leak persistence in emphysematous lungs with reduced parenchymal elastic recoil. Conversely, the absence of external suction allows the visceral and parietal pleura to appose under physiologic conditions, favoring fibrin deposition and closure of small alveolar leaks. These mechanisms provide a coherent biological rationale for the shorter chest tube duration observed in the No Suction arm and are consistent with prior physiological and clinical observations.

The study findings are consistent with most prior RCTs, which showed either no difference or a favorable effect of water seal on air leak duration [14]. In the current study, suction did not help seal leaks faster. The No Suction group’s leaks stopped sooner on average; hence, we concur that suction should be reserved for large, clinically significant leaks or if the lung is not fully expanded (to avoid pleural space).

The current study reinforces other RCTs observing the lack of benefit for routine suction, e.g., in patients after lobectomy or wedge resection who were randomized to low-pressure suction vs. no suction from the time of surgery, adding suction made no difference in terms of air leak resolution [25,28].

A few studies have advocated for suction [6]. Some surgeons applied suction for 24–48 h post-surgery, fearing that without it, air or fluid might accumulate. Nonetheless, the cumulative evidence does not support routine use.

The current protocol monitored if a patient on water seal had any sizeable pneumothorax or worrisome leak, then intervention with suction was done. The majority of patients did well without suction, and imaging was carefully evaluated to ensure lung expansion was adequate.

The ESTS/ERAS 2019 guideline recommends that chest drains be placed to water seal after anatomic lung resection, without external suction, barring specific reasons to use it [9]. Similarly, the STS 2024 consensus on pleural drains emphasizes individualized use of suction to be applied selectively, for instance if there is incomplete lung expansion or a significant ongoing air leak with suboptimal drainage [17]. The present study provides evidence in line with these recommendations, that tailored chest tube management improves outcomes. Most patients can be managed with a simple water seal (or digital equivalent) and will have their CTR sooner, whereas patients with large air leaks can be promptly identified (especially with digital monitors) and managed with suction or additional interventions as needed.

In our study, although the primary outcome of CTD was significantly shorter in the No Suction compared to the Suction group, it did not translate to a shorter LOS of the No Suction group. This finding may be explained by the understanding that patient hospital discharge is influenced by many factors in which ICD is only one of them.

One strength of the current study was the use of a DDS for all patients, which improved the accuracy of leak detection and the decision-making for CTR. DDS provide objective measurements of air leak flow, allowing clinicians to CTR even if a minimal intermittent air leak was present, as long as the leak flow is below a safe threshold (often <20 mL/min) [30]. Traditional water seal assessment can be subjective, possibly leading to more cautious delays in removal. By using the Thopaz device set to −8 cmH_2_O for water seal, the baseline negative pressure across patients was effectively standardized. Research on digital vs. analog systems [5] speculate that DDS are associated with shorter CTD and LOS, because they apply a mild regulated suction (e.g., −8 cmH_2_O) as physiologic suction [25]. In the present study, the Suction group patients were on digital suction rather than unregulated wall suction during the first night. All patients had a regulated system limiting excessive negative pressure. This could explain why the observed significant differences are somewhat modest (13 h) compared to other studies where wall suction might have had a more drastic effect [31]. The DDS likely prevented extremely high suction levels and thus perhaps mitigated some potential harm of suction. The current study noted a clear difference favoring no suction, which underscores that even a moderate −20 cmH_2_O overnight is unnecessary for the majority of patients.

Another important aspect is patient-centered outcomes such as pain and mobility. Early CTR has been associated with better patient-reported comfort and does not increase complications when done based on appropriate criteria [32]. While this study did not formally track pain scores, it aligns with ERAS principles of minimizing invasive supports as early and as safely possible to facilitate recovery [9].

Areas of disagreement or caution in the literature primarily revolve around specific scenarios rather than the routine case. One scenario is if a patient has a large PAL, some surgeons suggest suction is needed to prevent pneumothorax and to maintain apposition of pleura [33]. In post-pneumonectomy patients, many surgeons avoid suction entirely to prevent shifting mediastinum [34]; however, pneumonectomy cases were not part of this study. Additionally, air leak grading can guide management: Cerfolio’s air leak score [35] or digital quantification can identify leaks unlikely to seal without intervention. If an air leak is extremely large (e.g., >500 mL/min on digital), suction alone may not help and surgical or other interventions should be considered [36]. Conversely, for smaller leaks, a water seal is sufficient and preferable [37].

The current study stratified analysis confirmed known risk factors (lung disease, extent of resection) for prolonged drainage showing that the benefit of omitting suction was present across all patient subgroups. It indicates that even in high risk-patients (with COPD or after lobectomy) routine suction did not hasten CTR or reduce PAL. On the contrary, in a patient with fragile emphysematous lungs (COPD), applying wall suction could risk enlarging a tear or causing subcutaneous emphysema; water seal is gentler on the lung [38]. The current data support the notion that routine suction is not justified even in higher-risk cases, and management should be individualized rather than reflexive.

The present study results and existing literature [37,39], advocate for a change in standard practice toward use of water seal (no suction) after lung resections.

This study’s strengths are the randomized design with stratification improving comparability between groups; use of a DDS ensuring objective measurement; and the large sample size providing adequate power to detect differences in the primary outcome.

This study has some limitations. First, the study was unblinded—surgeons and patients knew which chest tube management was being used. However, the endpoints (time to removal, occurrence of PAL) are objective and were determined by protocol criteria. There is a possibility that knowing a patient is on suction could influence the threshold for removing a tube, whereas with digital monitoring on water seal, one might remove earlier. An attempt to mitigate was done by using objective digital criteria for all patients. Second, the study’s protocol involved suction only for the first night in the Control group, which is a relatively short duration of suction. The present results specifically address the value of an initial 12–24 h of suction. It cannot exclude that suction applied for different durations might have other effects. Third, the study population was predominately lung cancer resections performed exclusively via VATS, which may limit generalizability to settings with longer hospitalizations, to open thoracotomy patients, or to patients with different profiles. Fourth, several intraoperative variables known to influence postoperative air leak and chest tube duration—including intraoperative air leak severity, fissure completeness, presence of pleural adhesions, and the use of intraoperative sealants—were not systematically recorded as part of the study protocol and therefore could not be included in the analysis. Although randomization with stratification by previous lung disease, BMI, and resection type was designed to balance these factors between groups, residual confounding cannot be entirely excluded. The exclusive use of VATS in all patients eliminates surgical-approach heterogeneity as a source of variability, but the inability to adjust for the aforementioned intraoperative variables remains a methodological limitation. Fifth, the observed standard deviations for time to chest tube removal (43.8 h in the No Suction group and 67.8 h in the Control group) substantially exceeded the 24 h value used in the a priori sample size calculation. This greater-than-anticipated dispersion was driven by a small subset of patients with prolonged air leak in each arm. Although the primary endpoint nonetheless reached statistical significance (Wilcoxon rank-sum test, *p* = 0.002), the analysis of prolonged air leak as a binary outcome may have been underpowered to detect a clinically relevant difference between groups, raising the possibility of a type II error. The numerically lower incidence of PAL in the No Suction group (3.6% vs. 8.1%) should, therefore, be interpreted with caution and warrants confirmation in a larger, adequately powered trial. Sixth, the analysis followed a per-protocol rather than an intention-to-treat approach: patients in the No Suction group who developed clinically significant air leak with lung collapse or massive subcutaneous emphysema and required suction or an additional drain were excluded from the analysis. This pre-specified strategy was chosen to evaluate the chest tube management protocols as actually delivered and was permitted by the protocol; however, it may have introduced bias in favor of the No Suction group. Given the small number of crossover events (less than 10% of the No Suction group), and the fact that all crossover patients exhibited clinically apparent indications for additional drainage, we believe the practical impact on the conclusions is likely modest, but residual bias cannot be excluded. A pre-specified intention-to-treat (ITT) sensitivity analysis was therefore performed (see Section 2.5), in which all 309 randomized patients were analysed in their original allocation arm regardless of subsequent cross-over or escalation of treatment; the direction of the effect and the overall conclusion of the per-protocol analysis were preserved in this ITT analysis, supporting the robustness of the findings. Finally, while the study focused on air leak-related outcomes, it did not formally measure pain scores or patient satisfaction, which would have been interesting to correlate with earlier CTR. Future studies could assess quality-of-life metrics to further document the benefits of avoiding suction.

## 5. Conclusions

In conclusion, this study provides robust evidence that routine postoperative ICD suction is not necessary for the majority of lung resection patients. Omitting suction leads to earlier CTR without increasing PAL or hospital stay. Surgeons should consider managing ICD on water seal by default and individualizing suction application based on the patient’s clinical situation. Embracing this strategy can contribute to enhanced recovery protocols by minimizing unnecessary interventions, reducing CTD, and potentially lessening pain and hospitalization for the patients. Future research may focus on optimizing criteria for CTR (leveraging digital metrics) and on adjunct methods to manage the occasional PAL.

## Figures and Tables

**Table 1:** Demographic characteristics of the study participants (values are given as *n*/N (%) or as specified).

Characteristic	Overall Cohort *N* = 286	Suction *N* = 149	No Suction *N* = 137	*p*-Value
Gender ^1^				0.6
Female	136/286, (48%)	68/149, (46%)	68/137, (50%)	
Male	150/286, (52%)	81/149, (54%)	69/137, (50%)	
Age (years)				0.7
N Non-missing (missing)	283 (3)	146 (3)	137(0)	
Mean (SD)	67.0 (13.2)	67.0 (13.8)	67.0 (12.6)	
Median (Q1, Q3) (Min, Max)	70.0 (62.0, 75.0) (0.0, 89.0)	71.0 (62.0, 75.0) (0.0, 89.0)	70.0 (63.0, 75.0) (0.0, 85.0)	
Smoking History	185/284, (65%)	95/147, (65%)	90/137, (66%)	0.9
Active Smoker ^1^	99/178, (56%)	55/93, (59%)	44/85, (52%)	0.3
(Missing)	108	56	52	
pack/year				0.8
N Non-missing (missing)	157 (129)	82 (67)	75 (62)	
Mean (SD)	50.9 (29.5)	51.9 (31.4)	49.9 (27.5)	
Median (Q1, Q3) (Min, Max)	50.0 (30.0, 60.0) (2.0, 150.0)	50.0 (30.0, 60.0) (2.0, 150.0)	50.0 (30.0, 60.0) (7.0, 120.0)	
Surgery for lung cancer ^1^	265/286, (93%)	136/149, (91%)	129/137, (94%)	0.4

^1^ *n*/*N* Non-missing, (percent), where *N* is non-missing sample size for that variable. Missing data were due to incomplete records.

**Table 2:** Clinical data.

Resection Type ^1^	Overall Cohort *N* = 286	Suction *N* = 149	No Suction *N* = 137	*p*-Value
Wedge resection (non-anatomical)	139/286, (49%)	72/149, (48%)	67/137, (49%)	>0.9
Anatomical segmentectomy	25/286, (8.7%)	15/149, (10%)	10/137, (7.3%)	0.4
Lobectomy	118/286, (41%)	60/149, (40%)	58/137, (42%)	0.7
Bi lobectomy	6/286, (2.1%)	3/149, (2.0%)	3/137, (2.2%)	>0.9

^1^ *n*/*N* Non-missing, (percent), where *N* is non-missing sample size for that variable. Missing data was due to incomplete records.

**Table 3:** Stratification factors.

Characteristic	Overall Cohort *N* = 286	Suction *N* = 149	No Suction *N* = 137	*p*-Value
Stratification				
Previous lung disease	57/286, (20%)	29/149, (19%)	28/137, (20%)	0.8
BMI < 22	42/286, (15%)	22/149, (15%)	20/137, (15%)	>0.9
Anatomical surgery	147/286, (51%)	77/149, (52%)	70/137, (51%)	<0.9

**Table 4:** Primary and secondary endpoints of the study.

Characteristic	Overall Cohort *N* = 286	Suction *N* = 149	No Suction *N* = 137	*p*-Value
Primary endpoint				
Time to drain removal (hours)				0.002
Mean (SD)	47.2 (57.9)	53.6 (67.8)	40.2 (43.8)	
Median (Q1, Q3) Range (Min, Max)	24.6 (18.4, 45.7) (10.7, 547.1)	25.9 (20.2, 52.3) (14.5, 547.1)	22.9 (16.5, 44.0) (10.7, 265.0)	
Secondary endpoints				
Length of hospital stay				0.13
Mean (SD)	2.7 (2.7)	2.9 (2.9)	2.6 (2.5)	
Median (Q1, Q3) Range (Min, Max)	2.0 (1.0, 3.0) (1.0, 20.0)	2.0 (1.0, 4.0) (1.0, 20.0)	2.0 (1.0, 3.0) (1.0, 16.0)	
Prolong Air leak	17/286, (5.9%)	12/149, (8.1%)	5/137, (3.6%)	0.12

**Table 5:** Overall effect of active suction on the study outcomes (regression estimates).

Drain	Removal	Time		LOS			PAL	
Beta	95% CI	*p*-Value	Beta	95% CI	*p*-Value	OR	95% CI	*p*-Value
13	0.03, 27	0.05	0.34	−0.29, 0.96	0.3	2.31	0.83, 7.43	0.12

Notes: Positive beta values indicate longer time or greater number of days associated with suction. OR > 1 indicates higher odds of prolonged air leak with suction.

**Table 6:** Regression analysis. The effect of stratification factors: previous lung disease, BMI, and anatomical surgery on outcomes.

	Drain	Removal	Time		LOS			PAL	
Predictor	Beta	95% CI	*p*-Value	Beta	95% CI	*p*-Value	OR	95% CI	*p*-Value
Suction (no lung disease)	11	−3.5, 26	0.14	0.23	−0.46, 0.92	0.5	3.31	0.78, 22.6	0.14
Previous lung disease	22	−1.2, 46	0.063	0.9	−0.21, 2.0	0.11	6.42	1.01, 50.7	0.048
Suction and previous lung disease	13	−20, 46	0.4	0.59	−0.96,2.1	0.5	0.52	0.05, 4.73	0.6
Suction (BMI ≥ 22)	28	−6.7, 63	0.11	0.57	−1.1, 2.2	0.5	4.22	0.56, 86.9	0.2
BMI < 22	5.7	−22, 33	0.7	0.74	0.54, 2.0	0.3	0.67	0.09, 13.5	0.7
Suction and BMI < 22	17	−20, 55	0.4	0.28	1.5, 2.0	0.8	0.45	0.02, 5.03	0.5
Suction (wedge non-anatomical)	22	3.1, 41	0.023	0.8	−0.07, 1.7	0.073	7.11	1.22, 135	0.07
Anatomical surgery	28	9.1, 47	0.004	1.7	0.85, 2.6	<0.001	4.0	0.57, 79.4	0.2
Suction and anatomical surgery	−17	−44, 9.4	0.2	−0.92	−2.1, 0.3	0.14	0.16	0.01, 1.6	0.2

Notes: Linear regression on the natural-log-transformed time to chest tube removal (hours) and gamma generalized linear model with a log link for length of stay (days); coefficients are reported on the original scale for ease of interpretation. Logistic regression for prolonged air leak (binary outcome). Reference categories: no previous lung disease, BMI ≥ 22 kg/m^2^, non-anatomical (wedge) resection, and No Suction group. Rows beginning with “Suction × \u2026” are the corresponding two-way interaction terms. Positive beta values indicate longer time or greater number of days in the Suction group relative to the No Suction reference within the indicated subgroup. OR > 1 indicates higher odds of prolonged air leak with suction. Wide confidence intervals around several PAL estimates reflect sparse-data instability driven by the low total number of PAL events (*n* = 17) and should be interpreted with caution.

## Data Availability

The data supporting the findings of this study are available from the corresponding author upon reasonable request.

## Abbreviations

The following abbreviations are used in this manuscript:

BMI	Body Mass Index
CI	Confidence Interval
COPD	Chronic Obstructive Pulmonary Disease
CTD	Chest Tube Duration
CTR	Chest Tube Removal
DDS	Digital Drainage System
ERAS	Enhanced Recovery After Surgery
ESTS	European Society of Thoracic Surgeons
ICD	Intercostal Drain/Chest Tube
LOS	Length of Stay
OR	Odds Ratio
PAL	Prolonged Air Leak
POD	Postoperative Day
RCT	Randomized Control Trial
SD	Standard Deviation
STS	The Society of Thoracic Surgeons
